# Consciousness across Sleep and Wake: Discontinuity and Continuity of Memory Experiences As a Reflection of Consolidation Processes

**DOI:** 10.3389/fpsyt.2017.00159

**Published:** 2017-09-07

**Authors:** Caroline L. Horton

**Affiliations:** ^1^DrEAMSLab, Psychology, Bishop Grosseteste University, Lincoln, United Kingdom

**Keywords:** sleep, dreaming consciousness, autobiographical memory, memory consolidation, continuity theory

## Abstract

The continuity hypothesis (1) posits that there is continuity, of some form, between waking and dreaming mentation. A recent body of work has provided convincing evidence for different aspects of continuity, for instance that some salient experiences from waking life seem to feature in dreams over others, with a particular role for emotional arousal as accompanying these experiences, both during waking and while asleep. However, discontinuities have been somewhat dismissed as being either a product of activation-synthesis, an error within the consciousness binding process during sleep, a methodological anomaly, or simply as yet unexplained. This paper presents an overview of discontinuity within dreaming and waking cognition, arguing that disruptions of consciousness are as common a feature of waking cognition as of dreaming cognition, and that processes of sleep-dependent memory consolidation of autobiographical experiences can in part account for some of the discontinuities of sleeping cognition in a functional way. By drawing upon evidence of the incorporation, fragmentation, and reorganization of memories within dreams, this paper proposes a model of discontinuity whereby the fragmentation of autobiographical and episodic memories during sleep, as part of the consolidation process, render salient aspects of those memories subsequently available for retrieval in isolation from their contextual features. As such discontinuity of consciousness in sleep is functional and normal.

## Continuity in Its General Form

The continuity hypothesis (1) posits that there is continuity, of some form, between waking and dreaming. A recent body of work [e.g., Ref. (1–10)] has provided convincing evidence for the characteristics of specific aspects of continuity, for instance that some salient experiences from waking-life feature in dreams over others. Five factors have been proposed to account for the incorporation of waking-life experiences into dreams (11): the emotionality of the waking-life experience; the type of waking-life activity; personality traits; time intervals; and time of night. Emotional arousal consistently seems to accompany these experiences, both during waking (original experience) and while dreaming [for a review, see Ref. (10)].

Continuity between dreaming and waking, therefore, appears to provide a framework for studying and measuring the extent of overlap, or shared variance, between different states of consciousness (11). This holds appeal in that—hypothetically—scholars could build models predicting dream content and characteristics given knowledge about specific predictor parameters, such as the emotionality of past experiences and time spent asleep. Previous attempts to conceptualize variability in dream behaviors such as dream recall (12) left 91.6% variance unexplained. As such the task at hand is ambitious. It also assumes that any as yet explained variance may be problematic, or simply, “error.” Indeed, discontinuities have been somewhat dismissed as being either a product of activation-synthesis [for instance, see Ref. (13)], an error within the consciousness binding process during sleep, a methodological anomaly, or simply as yet unexplained. Furthermore, the concept of continuity remains somewhat ill-defined (14). This paper presents a conception of discontinuity within dreaming and waking cognition for the first time, arguing that disruptions of consciousness are as common a feature of waking cognition as of dreaming cognition, and that processes of sleep-dependent memory consolidation of autobiographical experiences can in part account for many of the discontinuities of sleeping cognition in a functional way. By drawing upon evidence of the incorporation, fragmentation, and reorganization of memories within dreams, this paper proposes a model of the fragmentation of autobiographical and episodic memories during sleep, which manifest as discontinuity during sleep, rendering salient aspects of those memories subsequently available for consolidation in isolation from their contextual features.

In sum the discontinuities of cognition in dreams have previously been recognized as being both typical of normal sleep mentation behavior, and possibly indicative of problematic dissociative symptoms, such as schizophrenia [e.g., Ref. (15, 16)]. This paper summarizes the elements of continuous and discontinuous processing across sleep and wake, recognizing that discontinuities in form, of cognition and of consciousness are typical and indeed functional of waking processing as well as in dreams (i.e., sleeping processing). At first glance, this may appear to be at odds with literature and evidence demonstrating that dreaming cognition, as a reflection of rapid eye movement (REM) sleep (13, 17), is dissociative and, therefore, symptomatic of psychosis. However, this paper will conceptualize discontinuity independently from concepts of continuity and dissociation in order to clarify this standpoint.

The continuity and discontinuity of dreaming mentation has to date been explored in a handful of studies, with the latter in part being related to the experience of bizarreness. This paper provides an alternative account of such discontinuities and attempts to conceptualize dreaming cognition as being functionally discontinuous for the first time, while offering theoretical accounts of continuity and discontinuity across sleep and wake.

There has thus far been little agreement over a definition of the term, “continuity,” when it refers to the carryover of aspects of waking life into dreams and dreams into waking life (14). This paper attempts to clarity this and proposes a workable conception of continuity, with reference to the retrieval of prior experiences into consciousness at any given time.

## Continuity of Consciousness

The field of sleep science well recognizes that sleep is a heterogeneous state. As distinct stages of sleep are defined by specific patterns of neural activity, measured using electroencephalogy, we assume that cognition during sleep changes also. What is less clear is how consciousness, usually measured using sleep or dream “mentation” reports, changes in line with this. This is less clear because it is difficult to access reports of conscious experience from sleep, as recall of those experiences is poor and changeable as an individual wakes from sleep.^1^ Perhaps, due to this methodological challenge, there has been relatively little interest in consciousness during sleep within the field of sleep science. Dream researchers, on the other hand, use a variety of methods to capture fragments of conscious experience from sleep, including at times relying on spontaneous awakenings or the “most recent dream” method of recall and report, which likely bias findings to a sample of REM sleep dreams. Dreams sampled from REM are, indeed, typically easier to recall than dreams from other stages of sleep (18) and are also more bizarre, lengthy and emotionally intense. Comparable changes have also been documented from dreams sampled across the night, shifting from early (more coordinated) to late (more bizarre and discontinuous) (7, 19, 20). This seems somewhat different to the traditional view of waking conscious thought as focused, under control, and efficient. To illustrate this, Hartmann (21) depicted a model of consciousness as a dimension ranging from focused thought in wake at one end, to looser, hyperassociative cognition in REM sleep at the other, with a variety of intermediate states such as daydreaming and non-REM sleep featuring some degree of both control and bizarreness.

However, it seems vastly over-simplistic to think of waking consciousness as being homogeneous. It also seems somewhat optimistic to characterize waking thought as consistently focused and efficient. Take for example the metacognitive awareness of realizing that you have been trying to read the same paragraph of text for some time, without really having processed any of it. Or the conscious act of daydreaming: a part-focused activity perhaps with a pleasant goal but one that requires fantasy, mind-wandering and a loss of engagement with reality. Other examples combining high-level metacognitive functioning with a loss of engagement with reality include experience deja-vu, having involuntary autobiographical memories (AMs) interrupt focused thought, and meditation. During wake, we often appear not to notice events in our immediate environment (for a more dramatic example consider change blindness, in which observers often fail to notice major visual changes to a stimulus). As such, consciousness is extremely discontinuous, particularly with its focus sometimes being on the external environment and sometimes detaching from this almost completely, with the conscious awareness being directed toward internal states of reflection. Furthermore, most theories of consciousness distinguish between the more essential and automatic processes of responding to environmental needs, and the high-order cognitive processes of awareness of self in place and time [e.g., Ref. (22, 23)].

While there seems to be change in the manifestation and perhaps function of consciousness both across sleep and wake, and within each of those states, there also exists continuity. Continuity across sleep and wake to some extent is unsurprising as the same neurophysiological and cognitive system (i.e., the person) functions across these states. Across wake, dreaming and daydreaming, for example, we are also able to recall our past experiences, to demonstrate awareness of moments when we somewhat lose engagement with reality and transport our present conscious awareness to the future in the form of planning and setting goals. Thus, consciousness in part involves the activation and awareness of memory. Memories for our own personal experiences, AMs (24–26) may provide a framework for such continuity over time. Indeed, the AM system acknowledges that a personal life story changes over time, with memories for specific experiences being updated and constructed in accordance with long-term goals and identities (25).

One way of conceptualizing continuity then is in terms of the activation of memories across time. Indeed, a dream that is continuous with waking life should, in theory, feature real experiences, or AMs. Continuity, therefore, would be high if AMs were activated and recalled accurately and in full. Conversely discontinuity, or low continuity, would prevail if AMs were activated partially or inaccurately. The problem with this view is that very rarely are AMs recalled in full and accurately. To recall an experience fully, with many details as featured in the original experience, would be to recall an episodic memory (24, 27, 28). Very rarely do we recall episodes in this way. Rather, we remember part-details and at times fill in the blanks, creating a narrative that aligns well with a goal or identity (26). Furthermore, this seems characteristic of sleeping mentation as well as wake (8, 29). As such, we can present two arguments: (i) consciousness is varied across both sleep and wake, yet (ii) in both states continuity of memory may not prevail.

Previous studies have explored the overlap, or continuity, of cognitive processing style from waking life to dreaming. Hobson et al.’s (13) activation-input-modulation (AIM) model of dreaming assumes that dreaming is illogical and lacks self-reflective awareness and directed thought, and it points to neurophysiological changes in REM sleep as the correlate for that mode of thought (30). However, empirical explorations of the continuity of cognition across sleep and wake have reflected more similarities than differences in cognition and metacognition (31), on measures of internal commentary, public self-awareness, frequency of emotion, self-reflection, and thwarted intentions. The only measure of cognition on which the two states differed were choice, event-related self-reflection and affect, with dreams being more emotional but having fewer feelings of volition, suggesting that dreaming and waking cognition are largely comparable in many respects (31). It is widely reported that the dorsolateral prefrontal cortex is relatively attenuated during REM sleep (32–34), which may well account for the reduced volition during REM sleep. However, we must be careful not to over-simplify these findings, recognizing that volition is still possible during sleep, as evidenced by the wealth of empirical work into the experience of lucid dreaming. Taken together, there is much evidence for comparable cognitive processing across sleep and wake. Kahan and LaBerge (35) suggest that differences between waking and dreaming cognition may be quantitative; there being more of certain cognitions in waking than dreaming and *vice versa*, but not qualitative, since there were incidences of the different types of cognitions sampled from both states.

Another way of accounting for the processing styles across wake and dreaming has been evidenced by Kahn and Hobson (36), who distinguished between thinking within dreams, which they argue was similar to waking thought states, and thinking about dreams, which they argued differed. Here, the authors compared cognitive functioning in dreams with that of wake, by asking participants questions about specific dream experiences. They highlighted that thinking within the dream is similar to thinking when awake even though some implausible activities and events within dreams are accepted without question (“thinking *about* …”), whereas in wake we are aware of the source of events. This awareness of the origin of events as being either internal, such as a thought or a dream, or external in that it is truly perceived, is known as reality monitoring (37). Reality monitoring is known to be deficient during dreaming, when we accept surrounding circumstances without question, with the exception of experiencing lucidity, when we become aware that we are dreaming (36, 38). Nevertheless we have further evidence that dreaming and waking cognition is largely comparable, with dreaming reflecting a sleep state in which reality monitoring is typically reduced. This gives rise to the possibility that the dreamer can process, or think about, scenarios that would not be encountered in waking life, perhaps as a rehearsal strategy of some kind [e.g., Ref. (39)].

This view is reiterated by Kozmová and Wolman (40), who point out that the differences between the findings of cognitive researchers and neurophysiological researchers may be explained by the fact that while Kahan and colleagues are focused on cognition within a dream, Hobson and colleagues in theories like the AIM model of dreaming focus more on features of dreaming such as uncertainty about people and places, and incongruous imagery. Nevertheless, the specific features of dreams as compared to waking memories may reflect the cognitive processing capacities at the time of being experienced and recalled, and consequently it is difficult to separate out cognition from features. In their study, Kozmová and Wolman (40) identified thought units as featuring cognitive or metacognitive processing, such as being analytical or operational, and in doing so illustrated the complexity of cognitions available in dreaming, concluding that the content of the dream (be it bizarre or not) does not preclude the application of rational thought, much like one would in waking life, and in fact the thought processes applied to a dream situation have much in common with those applied to a situation experienced in waking life. Any differences in cognitions between sleep and wake may be exemplified by the generation of bizarre scenarios, than how the mind approaches those scenarios, in line with Kahn and Hobson (36) views.

Kahn and Hobson (36) further account for the lack of reality monitoring during REM sleep dreaming by the reduced activity of the dorsolateral prefrontal cortex and precuneus, as well as the functional disconnect between these structures (32–34), meaning the brain cannot distinguish between who, where and what we are within a given context. This can account for not only the poor reality monitoring during dreaming, but also the typically disorganized or bizarre string of memory sources that comprise our dreams (41). As such, the way in which our cognition operates across different states may be largely continuous, but there seems to exist differences in the *organization* of our memories between sleep and wake. Given the complexities of the characteristics of continuity between sleep and wake and even across sleep (7, 19), it is unsurprising that efforts to account for continuity have thus far been limited.

## What Might be the Function of Continuity?

If we proceed with our definition of consciousness as a holistic experience of the present, brief past, long-term past (*via* memory retrieval) and the future (goal directed), we may assume that the ability to recall prior experiences, i.e., for continuity to function, would allow focus to be maintained in reaching future goals. In addition, as with the function of any memory system, the ability to recall prior experiences is the consequence of successful learning and allows those experiences to shape our interaction with the world in light of them. The activation of prior experience in a truly continuous manner would mean that we need to access all aspects of that prior experience. As we have seen, prior experiences are rarely recalled episodically, and so perhaps only salient aspects are particularly accessible at any one time, depending on need. Similarly, during sleep prior experiences may be activated either in full [rarely; see Ref. (8)] or in part and the activation of those salient aspects may strengthen them for future accessibility (42).

Ordered, controlled, and focused consciousness is also deemed to be a marker of psychological health, with authors such as Llewellyn (16) suggesting that a less structured or continuous consciousness may reflect elements of psychosis. Psychosis is characterized by disorganized cognition, seemingly leaping from one thought to another. The breakdown of holistic conscious experience is also reflected in dissociation; detachment from either the perception of reality or separation of the experience of the past. The essential feature of Dissociative Disorders is defined as “a disruption of and/or discontinuity in the normal integration of consciousness, memory, identity, emotion, perception, body representation, motor control, and behaviour” (43). The DSM-5 positions the dissociative disorders next to the trauma- and stressor-related disorders, to reflect the close relationship between these diagnostic categories (44). Dissociation can be exacerbated by sleep loss (45) and sleep disturbances are symptomatic (or perhaps even causal) of virtually all mental disorders as listed in the DSM-5 (43). This implies that sleep serves an integrative function for the mind and body. Indeed, in cognitive terms during sleep, we activate and re-activate experiences from the past (in particular the recent past), giving rise to the experience of dreams when we can recall the mental activation upon awakening. However, as we have already seen, when we explore the characteristics of dreams it is evident that consciousness is rarely continuous [e.g., Ref. (7, 18)]. As such while sleep may help to maintain order of consciousness and overall mental health, it apparently does not reflect continuity of it.^2^

## What is Discontinuity?

If continuity refers to the ability to retrieve the past, and the future where relevant (goals), in the context of present awareness (consciousness), then it would follow logically that discontinuity is the opposite of this, whereby discontinuity would be the inability to retrieve the past or future in the context of the present. Also, if continuity involves recalling past experiences holistically, discontinuity could refer to recalling them in a fragmentary manner. As we have noted, such discontinuity seems characteristic of both waking and sleeping cognition.

However, discontinuity may not always be conceptualized in opposition to continuity. Both Hobson and Schredl (14) and Malinowski and Horton (5, 6) acknowledge that the continuity-discontinuity spectrum may be a gradient, rather than discrete entities. For example, recalling a dream (continuity of dreaming memories to wake) may involve accessing a number of features of the original experience, but with some added appraisal of the experience and with some features being omitted from memory. This appears both continuous and discontinuous at the same time. Similarly dreaming of an upcoming social event (from waking life) may seem largely continuous in the manner of reflecting concerns, characters involved and the general setting, but could be discontinuous in that the actual future event could never happen. Here, then dream–wake (or wake–dream) continuity would perhaps rarely be clearly either continuous or discontinuous, but comprising features of both.

This presents a challenge with conceptualizing continuity and discontinuity in operational terms. Future work should aim to characterize dream–wake continuity in specific ways, noting trends for specific memory features to be either largely continuous or discontinuous.^3^ This may help to alleviate concerns with discontinuity as being merely unexplained variance in models of continuity that aim to predict dreaming behavior.

As we have noted, continuity can take many forms. Discontinuity could present as disjointed cognition, with absences or lapses in attention (i.e., consciousness being interrupted). Alternatively discontinuity could present, in extreme cases, as dissociation in waking, or bizarreness in dreams (consciousness during sleep). REM dreams are typically bizarre (48, 49), featuring sudden changes and eliciting feelings of strangeness, curiosity or mystique upon awakening. As we have seen, dreams very rarely feature memories from waking life in a truly episodic form (8, 29) and so are, by definition, discontinuous. Indeed, it seems likely that the only instances in which memories are replayed in their precise manner as they were originally experiences, is when they are so emotionally intense and negatively valenced that they were traumatic to the individual (50). In sum, full continuity here may reflect a maladapted system, while discontinuity is the norm.

Indeed several fields of study indicate the central role of the emotionality of a (waking) experience as being important for its subsequent recallability (51, 52), consolidation [e.g., Ref. (53, 54)]; and, perhaps relatedly, its likelihood of being dreamed about [(55, 56); see also Ref. (10) for a review]. Furthermore, some studies have implied that there exists a relationship between continuity and emotional intensity, such that the more intensely emotional the experience, the more highly continuous it is, especially if it is traumatic in nature [e.g. Ref. (57), see also (58)]. A somewhat emotional experience will likely be dreamed about, but in a less continuous manner [e.g., see Ref. (7, 19, 55)]. An experience low in emotional intensity may be unlikely to require processing or be dreamed about (55). Thus, there may be just cause to further explore relationships between continuity and emotionality in dream studies. Indeed, in order to explore discontinuities across sleep and wake, Kunzendorf et al. (59) reported that dreams were more bizarre, more “dream-like,” and more emotional than daydreams. Hartmann et al. (60) found that the contextualizing images in dreams were more emotionally intense than those in daydreams, and also that dreams had mostly fear/terror and helplessness/vulnerability as their contextualizing emotion, whereas daydreams had no particularly dominant emotions. Thus, consciousness during sleep appears to be more bizarre and emotional than daydreams, although emotionality and bizarreness (perhaps representing discontinuity) is related across all states. It is also interesting to note that daydreams generated under the influence of extreme emotional intensity are more “dream-like” and more symbolic than any other kind of daydream (61). This again illustrates that the content of dreams may be different to waking thought, but, as above, the cognitions about that content may be more similar.

An interesting caveat to the generalization about dreams versus daydreams comes from Kunzendorf et al. (59) who found that participants with “thin boundaries,” i.e., people who blur the lines between various aspects of life (including waking and dreaming) more than those with “thick boundaries” who see things in more clear-cut, black-and-white terms, had daydreams that were equally as bizarre as nocturnal dreams, reinforcing the idea that there may exist strong individual differences between people who experience continuity and those who do not. The assumptions within this paper on the whole refer to patterns of general cognitive activity irrespective of any such trait effects. However, both the fields of dream science and psychopathology indicate that there exist differences between individuals in the extent to which they may experience continuity or not. For instance, some individuals are more susceptible to nightmares than others, which often feature more episodic incorporations of waking-life episodes (57). Furthermore and not unrelatedly, some individuals are more vulnerable to experiencing dissociation than others. This raises questions concerning the extent to which continuity and discontinuity may be “normal” in terms of being psychologically healthy. As stated earlier, continuity may reflect maladaptive processing. However, severe discontinuity, as in the case of experiencing dissociation, reflects a disorganized and interrupted sense of reality and as such is clearly maladaptive also. Thus, part of the role of characterizing the extent and frequency of experiencing discontinuity should help to address the issue of, “*how much discontinuity is normal*?”

## A Possible Function of Discontinuity?

If discontinuity is the norm, rather than indicating a disordered system as previous literature has implied, it may have a function. A number of theories of dreaming and of consciousness have attempted to account for discontinuity in the past, and a brief review of these will follow.

Hobson et al.’s (13) AIM model of dreaming focuses on dreaming occurring during REM, which has not been empirically supported as dreams can be sampled easily from non-REM sleep and REM does not always lead to dream recall. Nevertheless, the model proposes that with correct activation and cognitive input, dreaming will result. A dream report is deemed to be the result of a “synthesis” of activations, which taken together are neither meaningful nor functional. As such according to this model, discontinuity is the norm during REM sleep and is taken to reflect the lack of order and purpose of the epiphenomena of dreams.

Somewhat relatedly, models, such as Schredl’s (1, 62), attempt to characterize the likelihood of recalling dreams and even dreaming of specific material. However, unexplained variance remains so high (91.6%) that these models have many more factors yet to uncover in the pursuit of understanding the nature of dreaming. The as yet unexplained variance may reflect discontinuity, which is difficult to conceptualize let alone predict using existing methods and the continuity hypothesis as a theoretical underpinning (1), though of course this does not imply necessarily that such discontinuity is unimportant. Rather just that dream scientists have much work to do!

An alternative set of views concerns the style of cognitive processing that seems typical of REM, or late-night, sleep. As Hartmann (21) suggested, but did not test empirically, REM sleep cognition may be *hyperassociative*, in that the links from one activated thought to another are more tenuous than in waking. Indeed, this is one way of thinking about discontinuity as illogical or surprising links from one processing sequence to another. In cognitive psychology, this would represent a weak semantic association rather than a strong one, with the latter typically reflecting focused thought and goal-oriented processing. Hartmann, however, did not speculate as to what the function of such hyperassociativity may have been, although he was a psychoanalyst and so believed that dreams had a language that could be uncovered therapeutically. Implicit in that view was the notion that manifest dream content was merely only part of a dream’s make-up, meaning that some degree of interpretation was required on Hartmann’s part. Such a view is largely at odds with more contemporary approaches to dream science, in which memory sources of dreams are deemed to reflect more explicitly the activation of waking-life experiences that may be processed during sleep, perhaps as part of a memory consolidation process (2–4).

An alternative body of work from both the sleep and dreaming fields explore the creativity associated with REM sleep (in particular). The hyperassociative processing style, or “loose” connections in Hartmann (21) terms, provide a mental space for creativity and novel solutions to become conscious. A great deal of evidence shows the relationships between REM sleep and creativity [e.g., Ref. (63)]. However, evidence also demonstrates the relationships between creativity and dissociation, with the latter in part being increased with sleep disturbances, as we have already seen (64), reinforcing the complex relationships between psychopathology and creativity.

Nevertheless the relationship between REM sleep and novel ideas has been shown in a number of empirical investigations exploring not only creativity but also problem solving and insight. Thus there seems to be something about the activity in REM sleep, or at least following several iterations of preceding non-REM sleep cycles, that promotes the formation of new concepts and ideas. Merely re-activating previous experiences episodically, i.e., continuity, would not logically facilitate such novelty. Rather, then discontinuity may involve the reactivation of some elements of previous experiences, but perhaps in parallel with additional experiences or in quick succession to dissimilar, or weakly associated, experiences—we do not yet know whether such hypothesized hyperassociativity operates in parallel or sequentially. We may assume that discontinuity involves the activation of memory fragments taken from a number of memory sources that are bound together into a conscious uniform experience, as dream images seem to comprise such condensed imagery (4, 65). If this is the case discontinuity, comprising hyperassociative processing, could be evidenced by the analysis of the memory sources of dreams. For example, one dream image featuring several identified memory sources, such as a character that we recognize from recent waking life, a familiar place in which the dream is set, the content of a conversation in the dream, as well as additional specific features, such as objects, people, or activities, can each be rated in terms of the dreamer’s familiarity with them, their age or time since being experienced (if at all), as well as more general features such as counting the number of memory sources in dream images across the night or sampled from different stages of sleep. Llewellyn (65) illustrates this in detail with her “quicksand dream,” showcasing how various different elements of waking life had been recombined and synthesized into a single, composite dream event. Llewellyn postulated that this process in dreaming operates to create episodic “landmark junctions” at a neural level. However, such discontinuity could have many purposes, not least to decontextualize each of the composite memory sources, rendering each individual contribution memorable in its own right. Such studies of discontinuity could inform much about the activation of autobiographical experiences across different states of consciousness.

To this end, discontinuity seems to allow novel insights and creative possibilities. However, very few individuals wake from a dream experiencing a sudden moment of clarity or enhanced understanding into a problem. As such, we cannot assume that discontinuity functions to make revelations conscious. Rather, discontinuity in sleep, and likely in wake also though perhaps to a lesser extent, may reflect offline processing that maintains the organization and consolidation of experiences within a neurocognitive system that is constantly being updated. During wake the human brain perceives a great wealth of novel information that requires processing and integration with existing structures. Much of this information will be superfluous to requirements and processing will cease very shortly after sensation. However, some will be salient enough to require consolidation into long-term memory networks, to be accessible for future use and to require prior knowledge to be updated. Such consolidation and re-consolidation likely occur constantly, though sleep seems to be the time when much of this consolidation occurs (66–70). Furthermore, sleep appears to afford opportunities for the reorganization of memory networks (22, 65, 71, 72) and thus the integration of newer experiences into pre-existing cortical structures (70, 73–75).

In order to evidence the idea that discontinuity engenders consolidation and integration of novel experiences with more established memory networks, we should be able to identify a change in the organization and representation of memory sources over time, as they become increasingly integrated. This should be mapped on to changes in the continuity, or order, of the conscious manifestation of that experience. A handful of dream studies have been able to map the activation of a specific memory source over time, although unfortunately these studies are varied in their methodological approach. Some short-term approaches have looked at the organization of memory representations across the night, with early-night sleep being rich in non-REM, slow-wave sleep, and later night sleep being richer in REM (7). More recently, we (19) have also demonstrated changes in the continuity of memory representations across the night, with dreams being sampled from later night sleep being more bizarre yet more integrated with older memory sources. Thus, to evidence a change in the organization and representation of a memory over time we may expect to see a memory source, appearing in a dream in a decreasingly literal manner as it is consolidated. Some postulate that the representation may become increasingly metaphorical (10), although this is difficult to evidence empirically. Hopefully in the future sleep study technology will afford the more accurate monitoring of this with neural mapping of the activation of the original memory engram along with a representation of the extent to which it has been integrated into long-term cortical structures. At present, we can rely on subjective dream reports and an analysis of their memory sources.

Long-term approaches have typically explored emotionally charged experiences being incorporated into dreams and have documented corresponding changes in the characteristics of those emotional memory representations over time [e.g., Ref. (62, 66, 76)]. Convincing evidence of relations between emotional waking-life experiences and dreams comes from studies with victims of trauma [for reviews, see Ref. (10, 77)]. For example, individuals with direct military experience of war have direct, unambiguous, and sometimes apparently veridical dream incorporations of their experiences; civilians, on the other hand, have “indirect” or “symbolic” incorporations (78). Thus, it may be that the more traumatic the experience, the more direct the incorporation of the experience into dreaming, as is also the case with posttraumatic stress disorder symptomatology. Cartwright (76) also reports of her studies with divorcees, who dreamed of their divorce process over time, and the shift from dream reports being more accurately reflective of current concerns and experiences, to being more symbolically represented as the divorce process was appraised and comes to terms with by the dreamer.

Indeed, these emotional experiences may be particularly pertinent. Several theories of sleep-dependent memory processing emphasize the role of emotion, either in identifying a particular aspect of a memory to be processed (79) or in signaling heightened activity during the consolidation process in sleep (80). Stickgold and Walker (42) presented a “triage” model of sleep-dependent memory consolidation, whereby salient aspects of an experience are tagged as requiring processing during sleep. Emotional intensity likely acts as this triage indicator. Concurrently, a growing number of studies now indicate that emotional waking-life experiences are more likely to feature in dreams compared to neutral experiences (9, 19, 55, 62, 81).

The consequence here is that experiences that have been well integrated into a memory network and that have been processed emotionally, would not manifest in dreams in a manner that largely reflects their original experience in waking life. In this way, the more discontinuous the representation of the experience, the more integrated and perhaps the more accepted (emotionally) it has become. Thus discontinuity can reflect a healthy cognitive system, rather than being a sign of psychopathology, as has been implied in previous accounts of consciousness. This may appear counterintuitive but suggests that discontinuity is a functional end-point of a cognitive process of integration and consolidation.

## The Characteristics of Memory Experience Across Sleep and Wake

Horton and Malinowski (4) proposed a model of AM consolidation in sleep, whereby memories for waking experiences can appear in dreams in largely fragmentary forms and as such differ from conventional manifestations of episodic memory. AMs in dreams can be sampled from non-REM as well as REM periods, which contain fewer episodic references and become more bizarre across the night (41, 82). Salient fragmented memory features are activated in sleep and rebound with fragments not necessarily emerging from the same memory, thus de-contextualizing those memories and manifesting as experiences that differ from waking conceptions. The constructive nature of autobiographical recall further encourages synthesis of these hyperassociated images into an episode *via* recalling and reporting dreams. This model of sleep-dependent memory consolidation accounts for the discontinuity within REM sleep mentation, and perhaps in elements of non-REM sleep reports and in aspects of waking also, as the fragmentary activation of specific memories being coupled with other memory fragments, giving rise to a new image, illustrates discontinuity clearly. Here, we can synthesize the characteristics of discontinuity with the activation of fragments of memory sources in sleep.

In sum the discontinuities of cognition in dreams have been recognized as being both typical of normal sleep mentation behavior, and possibly indicative of problematic dissociative symptoms, such as schizophrenia [e.g., Ref. (16)]. In this paper, I summarized the elements of continuous and discontinuous processing across sleep and wake, recognizing that discontinuities in form, of cognition and of consciousness are typical and indeed functional of waking processing as well as in dreams (i.e., sleeping processing).

The behavioral outcome, i.e., what is consolidated and/or consciously experienced is, more often than not, unclear upon awakening and usually later following dreaming. As the dream memory fades so quickly and it is only in the case of the few avid dreamers who may engage in consideration of their dream experiences that they claim to have an understanding of what their dream “meant” or represented, we cannot assume that a clear purpose of dreaming is to have conscious clarity or insight following the dream experience. The discontinuity of a dream and the associated feelings of bizarreness may contribute to this perception that dreams are perhaps not there to be understood as such. Nevertheless, discontinuity within and across dreaming and waking may still be functional if the activated memory fragments in sleep, manifesting in dream reports, remain accessible over and above other, non-incorporated memories at a later date.

## Conclusion

Discontinuity as we have conceptualized it here is the fragmentation of experiences as we tend to perceive them in the present. That may refer to dreaming of an event from waking life in a very different way to the original experience, or it may refer to simply recalling an experience in a different way to its original occurrence. Thus, discontinuity could refer to the change of a memory representation over time, whether that be in a dream or when recalled at a later time in waking.

In this paper, I have argued that discontinuity is typical of both waking as well as sleeping cognition as in waking it is a normal characteristic of autobiographical remembering. During sleep, as evidenced from dream reports from across the night, experiences from our waking lives are particularly discontinuous as specific constituent features of autobiographical experiences are selected for processing. However, under the backdrop of continuous consciousness, whereby the stream of thoughts, images, and cognitions are sewn together into a coherent narrative, individual memory fragments are bound together to create a new “episode,” which is certainly discontinuous from waking life as it may be implausible, incongruous or impossible (e.g., dreaming of talking to someone who has recently passed away).

The consequence of this is that the dream product may provide insight via consideration of novel experiences, but may not have been composed in a deliberately metaphorical or symbolic manner.

This leads us to present some testable hypotheses for future work in this field, linking sleep-dependent memory consolidation, the characteristics of dreams (of prior waking experiences) and the recallability of AMs over time. Experiences from waking life (AMs) that have been dreamed about in a discontinuous manner would likely be less emotionally intense than those that are continuous. Also the memory fragments that were rebound with another memory (source) during dreaming should lead to enhanced recallability of both the original source of the memory fragment as well as the memory source that appeared in the dream, relative to memories and fragments that had not been dreamed about, due to all these aspects having been activated during sleep. A recent study (83), documented the incorporation of recent autobiographical experiences into dreams that had been documented in a 14-day diary. Memory for those original experiences was tested *via* recall and recognition. Those experiences that had been incorporated into dreams were better recalled and recognized later. While this may seem unsurprising as the act of reporting a dream could be seen to rehearse those original experiences, the representation of the experience in the dream was often far different to the original episode. This provides a starting framework for exploring links between (dis)continuity and memory consolidation of declarative experiences.

These predictions present exciting opportunities for the collaboration of dream and sleep scientists with memory consolidation scholars, to explore the nature of sleeping mentation as a reflection of the cognitive processes of memory reorganization over time. They also should not detract from the contributions and efforts of continuity theorists, who may, in part, aim to predict dream behaviors given certain parameters. Rather, discontinuity needs to be characterized in relation to continuity to help us to appreciate further the wonderful changing state of consciousness, what it might tell us about the activation of memories during sleep; something that neuroimaging methods are not yet able to do in a detailed manner.

Furthermore, scholars should continue attempting to identify whether patterns of discontinuity and continuity are consistent or varied across individuals. For instance, some existing work has identified benefits of a labile sleep–wake pattern in terms of the number of cycles through sleep stages upon knowledge awareness tasks (84, 85) and recent work has identified that lucid dreaming training benefits performance in field independence tasks (86). As such identifying patterns of cognitive functioning and discontinuity of consciousness that seem optimal for both mental health and memory consolidation could pave the way for similar training or intervention studies, if patterns of discontinuity may be changeable across individuals.

## Author Contributions

As sole author CH prepared the manuscript and ideas therein in full.

## Conflict of Interest Statement

The author declares that the research was conducted in the absence of any commercial or financial relationships that could be construed as a potential conflict of interest.

## Footnotes

^1^It is assumed, here, that dreaming is considered to be a reflection of conscious activity during sleep and that, as such, it is not different to sleeping cognition or even reflective of specific stages of sleep. Indeed, several studies demonstrate that dreams can be successfully sampled from sleep onset, non-REM as well as REM stages under appropriate systematic conditions [e.g., Ref. (18)]. As such, the definition of dreaming preferred here is *a report of a mental activity experienced during sleep*.

^2^It is necessary to mention here that in these discussions that acknowledge the changeable nature of consciousness, we are referring to continuous and discontinuous functioning as a state, rather than a trait. Explorations of dissociative tendency typically refer to individuals who experience discontinuity more readily and frequently than others. However, my own work has noted that, despite these individual differences, there exists great variability within people’s dreaming tendencies. As such experiencing a particularly discontinuous dream, for instance, should not necessarily indicate problematic cognition or experiences, especially of a clinical nature. Furthermore, therapists and clinicians should always be wary of relying on dreams as part of any clinical diagnosis, unless sampled reliably and systematically. Great biases exist in dream reports, with some individuals much more likely to recall dreams generally than others [e.g., see Ref. (46)]. As such, encouraging anyone to try to recall a dream could over-rely on creative or imaginative processes, rather than capturing a valid dream memory. Similarly, some individuals seemingly very ready to report a dream could be particularly susceptible to creating the report without apparent effort, falsely believing that their report is true (47).

^3^One present project within my own lab is exploring this, by creating a dream memory database and charting the likelihood of individual memory fragments (objects, characters, place, sounds, etc.) as being continuous or discontinuous.

## References

[1] Schredl M Hoffman F. Continuity between waking activities and dream activities. Conscious Cogn (2003) 12:298–308. 10.1016/S1053-8100(02)00072-7 12763010

[2] Horton CL Malinowski J Re-defining discontinuity: implications for the functions of dreaming. Int J Dream Res (2011) 4(2):78–80. 10.11588/ijodr.2011.2.9147

[3] Horton CL Malinowski JE Autobiographical and episodic memory sources of dreams. Int J Dream Res (2011) 4(Suppl 1):S44–5. 10.1111/jsr.12134 24635722

[4] Horton CL Malinowski JE. Autobiographical memory and hyperassociativity in the dreaming brain: implications for memory consolidation in sleep. Front Psychol (2015) 6:874. 10.3389/fpsyg.2015.00874 26191010 PMC4488598

[5] Malinowski JE Horton CL The sleeping therapist: do we dream to process emotional waking experiences? Int J Dream Res (2011) 4(Suppl 1):S48.

[6] Malinowski JE Horton CL Themes of continuity. Int J Dream Res (2011) 4(2):86–92. 10.11588/ijodr.2011.2.9149

[7] Malinowski J Horton CL Differences in dreams of waking life from early-night to late-night sleep. Dreaming (2014) 24(4):253–69. 10.1037/a0037817

[8] Malinowski JE Horton CL Memory sources of dreams: the incorporation of autobiographical rather than episodic experiences. J Sleep Res (2014) 23(4):441–7. 10.1111/jsr.12134 24635722

[9] Malinowski J Horton CL Emotion but not stress modulates the incorporation of waking experiences into dreams. Dreaming (2014) 24(1):18–31. 10.1037/a0036017

[10] Malinowski JE Horton CL Metaphor and hyperassociativity: the imagination mechanisms behind emotional memory assimilation in sleep and dreams. Front Psychol (2015) 6:1132 10.3389/fpsyg.2015.01132 26347669 PMC4539471

[11] Schredl M Continuity between waking and dreaming: a proposal for a mathematical model. Sleep Hypn (2005) 5(1):26–40.

[12] Schredl M Wittmann L Ciric P Götz S. Factors of home dream recall: a structural equation model. J Sleep Res (2003) 12(2):133–41. 10.1046/j.1365-2869.2003.00344.x 12753350

[13] Hobson JA Pace-Schott EF Stickgold R. Dreaming and the brain: toward a cognitive neuroscience of conscious states. Behav Brain Sci (2000) 23:793–842–1121. 10.1017/S0140525X00003976 11515143

[14] Hobson JA Schredl M The continuity and discontinuity between waking and dreaming: a dialogue between Michael Schredl and Alan Hobson concerning the adequacy and completeness of these notions. Int J Dream Res (2011) 4(1):3–7. 10.11588/ijodr.2011.1.9087

[15] Dresler M Wehrle R Spoormaker VI Steiger A Holsboer F Czisch M Neural correlates of insight in dreaming and psychosis. Sleep Med Rev (2015) 20:92–9. 10.1016/j.smrv.2014.06.004 25092021

[16] Llewellyn S If waking and dreaming became dedifferentiated, would schizophrenia result? Conscious Cogn (2011) 20(4):1059–88. 10.1016/j.concog.2011.03.022 21498086

[17] Paiva T Bugalho P Bentes C. Dreaming and cognition in patients with frontotemporal dysfunction. Conscious Cogn (2011) 20:1027–35. 10.1016/j.concog.2011.06.008 21737311

[18] Baylor GW Cavallero C. Memory sources associated with REM and NREM dream reports throughout the night: a new look at the data. Sleep (2001) 24(2):165–70. 11247052

[19] Malinowski J Horton CL Testing the emotion assimilation theory: the effects of emotional intensity and time of night on wake-dream continuity. Int J Dream Res (2016) 9(Suppl 1):S56.

[20] Nir Y Tononi G. Dreaming and the brain: from phenomenology to neurophysiology. Trends Cogn Sci (2010) 14:88–100. 10.1016/j.tics.2009.12.001 20079677 PMC2814941

[21] Hartmann E The dream always makes new connections: the dream is a creation, not a replay. Sleep Med Clin (2010) 5:241–8. 10.1016/j.jsmc.2010.01.009

[22] Cicogna PC Bosinelli M. Consciousness during dreams. Conscious Cogn (2001) 10:26–41. 10.1006/ccog.2000.0471 11273624

[23] Hobson JA. REM sleep and dreaming: towards a theory of protoconsciousness. Nat Rev Neurosci (2009) 10(11):803–13. 10.1038/nrn2716 19794431

[24] Conway MA. Sensory-perceptual episodic memory and its context: autobiographical memory. Philos Trans R Soc Lond B Biol Sci (2001) 356:1375–84. 10.1098/rstb.2001.0940 11571029 PMC1088521

[25] Conway MA Pleydell-Pearce CW The construction of autobiographical memories in the self-memory system. Psychol Rev (2000) 107(2):261–88. 10.1037/0033-295X.107.2.261 10789197

[26] Conway MA Loveday C Remembering, imagining, false memories & personal meanings. Conscious Cogn (2015) 33:574–81. 10.1016/j.concog.2014.12.002 25592676

[27] Tulving E Precis of elements of episodic. Behav Brain Sci (1984) 7(2):223–38. 10.1017/S0140525X0004440X

[28] Tulving E Episodic memory: from mind to brain. Annu Rev Psychol (2002) 53:1–25. 10.1146/annurev.psych.53.100901.135114 11752477

[29] Fosse MJ Fosse R Hobson JA Stickgold RJ. Dreaming and episodic memory: a functional dissociation? J Cogn Neurosci (2003) 15:1–9. 10.1162/089892903321107774 12590838

[30] Hobson JA Hoffman SA Helfand R Kostner D Dream bizarreness and the activation-synthesis hypothesis. Hum Neurobiol (1987) 6:157–64. 3449484

[31] Kahan TL LaBerge S Levitan L Zimbardo P. Similarities and differences between dreaming and waking cognition: an exploratory study. Conscious Cogn (1997) 6(6):132–47. 10.1006/ccog.1996.0274 9170565

[32] Braun AR Balkin TJ Wesenten NJ Carson RE Varga M Baldwin P Regional cerebral blood flow throughout the sleep-wake cycle. An H2(15)O PET study. Brain (1997) 120(Pt 7):1173–97. 10.1093/brain/120.7.1173 9236630

[33] Maquet P. Functional neuroimaging of normal human sleep by positron emission tomography. J Sleep Res (2000) 9:207–31. 10.1046/j.1365-2869.2000.00214.x 11012860

[34] Maquet P Phillips C. Functional brain imaging of human sleep. J Sleep Res (1998) 7(Suppl 1):42–7. 10.1046/j.1365-2869.7.s1.7.x 9682193

[35] Kahan TL LaBerge SP Dreaming and waking: similarities and differences revisited. Conscious Cogn (2011) 20(3):494–514. 10.1016/j.concog.2010.09.002 20933437

[36] Kahn D Hobson JA. State-dependent thinking: a comparison of waking and dreaming thought. Conscious Cogn (2005) 14(3):429–38. 10.1016/j.concog.2004.10.005 16091263

[37] Johnson MK Kahan TL Raye CL Dreams and reality monitoring. J Exp Psychol Gen (1984) 113(3):329–44. 10.1037/0096-3445.113.3.329 6237167

[38] Horton CL Conway MA Cohen G Memory for thoughts and dreams. 2nd ed In: Cohen G Conway MA, editors. Memory in the Real World. Hove: Psychology Press (2007). p. 281–304.

[39] Revonsuo A The reinterpretation of dreams: an evolutionary hypothesis of the function of dreaming. Behav Brain Sci (2000) 23:877–90. 10.1017/S0140525X00004015 11515147

[40] Kozmová M Wolman RN Self-awareness in dreaming. Dreaming (2006) 16(3):196–214. 10.1037/1053-0797.16.3.196

[41] Cavallero C Foulkes D Hollifield M Terry R. Memory sources of REM and NREM dreams. Sleep (1990) 13:449–55. 2287856

[42] Stickgold R Walker MP. Sleep-dependent memory triage: evolving generalization through selective processing. Nat Neurosci (2013) 16(2):139–45. 10.1038/nn.3303 23354387 PMC5826623

[43] American Psychiatric Association. Diagnostic and Statistical Manual of Mental Disorders. 5th ed Washington, DC: American Psychiatric Association (2013).

[44] Spiegel D Lewis-Fernandez R Lanius R Vermetten E Simeon D Friedman M. Dissociative disorders in DSM-5. Annu Rev Clin Psychol (2013) 9:299–326. 10.1146/annurev-clinpsy-050212-185531 23394228

[45] van Heugten-van der Kloet D Giesbrecht T Merckelback H. Sleep loss increases dissociation and affects memory for emotional stimuli. J Behav Ther Exp Psychiatry (2015) 47:9–17. 10.1016/j.jbtep.2014.11.002 25462597

[46] Beaulieu-Prévost D Zadra A. Absorption, psychological boundaries and attitude towards dreams as correlates of dream recall: two decades of research seen through a meta-analysis. J Sleep Res (2007) 16(1):51–9. 10.1111/j.1365-2869.2007.00572.x 17309763

[47] Horton CL Dream recall and confabulation. Imagination Cogn Pers (2014) 34(2):163–77. 10.2190/IC.34.2.e

[48] Strauch I REM dreaming in the transition from late childhood to adolescence: a longitudinal study. Dreaming (2005) 15(3):155–69. 10.1037/1053-0797.15.3.155

[49] Sutton JP Rittenhouse CD Pace-Schott E Stickgold R Hobson JA A new approach to dream bizarreness: graphing continuity and discontinuity of visual attention in narrative reports. Conscious Cogn (1994) 3(1):61–88. 10.1006/ccog.1994.1005

[50] Harvey AG Jones C Schmidt DA. Sleep and posttraumatic stress disorder: a review. Clin Psychol Rev (2003) 23:377–407. 10.1016/S0272-7358(03)00032-1 12729678

[51] Cahill L McGaugh JL. A novel demonstration of enhanced memory associated with emotional arousal. Conscious Cogn (1995) 4(4):410–21. 10.1006/ccog.1995.1048 8750416

[52] Hamann SB. Cognitive and neural mechanisms of emotional memory. Trends Cogn Sci (2001) 5(9):394–400. 10.1016/S1364-6613(00)01707-1 11520704

[53] Lewis PA Cairney S Manning L Critchley HD. The impact of overnight consolidation upon memory for emotional and neutral encoding contexts. Neuropsychologia (2011) 49(9):2619–29. 10.1016/j.neuropsychologia.2011.05.009 21621549 PMC7614373

[54] Nishida M Pearsall J Buckner RL Walker MP. REM sleep, prefrontal theta, and the consolidation of human emotional memory. Cereb Cortex (2009) 19:1158–66. 10.1093/cercor/bhn155 18832332 PMC2665156

[55] Horton CL Smith MD Proctor C The emotionality of dream memory sources: intensity and valence influences likelihood of incorporation. Int J Dream Res (2012) 4(Suppl 1):S45.

[56] Schredl M Doll E. Emotions in diary dreams. Conscious Cogn (1998) 7(7):634–46. 10.1006/ccog.1998.0356 9817817

[57] Levin R Nielsen TA. Disturbed dreaming, posttraumatic stress disorder, and affect distress: a review and neurocognitive model. Psychol Bull (2007) 133(3):482–528. 10.1037/0033-2909.133.3.482 17469988

[58] Nielsen TA Laberge L Paquet J Tremblay RE Vitaro F Montplaisir J. Development of disturbing dreams during adolescence and their relation to anxiety symptoms. Sleep (2000) 23(6):727–36. 10.1093/sleep/23.6.1 11007439

[59] Kunzendorf RG Hartmann E Cohen R Cutler J Bizarreness of the dreams and daydreams reported by individuals with thin and thick boundaries. Dreaming (1997) 7:265–71. 10.1037/h0094482

[60] Hartmann E Kunzendorf R Rosen R Grace N Contextualizing images in dreams and daydreams. Dreaming (2001) 11(2):97–104. 10.1023/A:1009488705828

[61] Hartmann E Kunzendorf RG Baddour A Chapwick M Eddins M Krueger C Emotion makes daydreams more dreamlike, more symbolic. Imagination Cogn Pers (2002) 22(3):257–76. 10.2190/TCFR-64LB-VGXF-A9FL

[62] Schredl M Factors affecting the continuity between waking and dreaming: emotional intensity and emotional tone of the waking-life event. Sleep Hypn (2006) 8:1–5.

[63] Cai DJ Mednick SA Harrison EM Kanady JC Mednick SC. REM, not incubation, improves creativity by priming associative networks. Proc Natl Acad Sci U S A (2009) 106(25):10130–4. 10.1073/pnas.0900271106 19506253 PMC2700890

[64] van Heugten-van der Kloet D Cosgrave J Merckelbach H Haines R Golodetz S Lynn SJ. Imagining the impossible before breakfast: the relation between creativity, dissociation and sleep. Front Psychol (2015) 6:324. 10.3389/fpsyg.2015.00324 25859231 PMC4374390

[65] Llewellyn S. Such stuff as dreams are made on? Elaborative encoding, the ancient art of memory, and the hippocampus. Behav Brain Sci (2013) 36:589–607. 10.1017/S0140525X12003135 24304746

[66] Cartwright RD. The role of sleep in changing our minds: a psychologist’s discussion of papers on memory reactivation and consolidation in sleep. Learn Mem (2004) 11:660–3. 10.1101/lm.75104 15576882 PMC534693

[67] Payne JD Ellenbogen JM Walker MP Stickgold R The role of sleep in memory consolidation. Learn Mem Compr Ref (2008) 2:663–85. 10.1007/7854

[68] Stickgold R Fosse R Walker MP Linking brain and behavior in sleep-dependent learning and memory consolidation. Proc Natl Acad Sci U S A (2002) 99(26):16519–21. 10.1073/pnas.012689199 12499518 PMC139175

[69] Walker MP. Sleep-dependent memory processing. Harv Rev Psychiatry (2008) 16(773557615):287–98. 10.1080/10673220802432517 18803104

[70] Walker MP Sleep-dependent memory integration. Front Neurosci (2009) 3(3):418–9.

[71] Landmann N Kuhn M Piosczyk H Feige B Baglioni C Spiegelhalder K The reorganisation of memory during sleep. Sleep Med Rev (2014) 18(6):531–41. 10.1016/j.smrv.2014.03.005 24813468

[72] Perogamvros L Dang-Vu TT Desseilles M Schwartz S. Sleep and dreaming are for important matters. Front Psychol (2013) 4:474. 10.3389/fpsyg.2013.00474 23898315 PMC3722492

[73] Axmacher N Draguhn A Elger CE Fell J. Memory processes during sleep: beyond the standard consolidation theory. Cell Mol Life Sci (2009) 66:2285–97. 10.1007/s00018-009-0019-1 19322518 PMC11115869

[74] Lee AK Wilson MA. Memory of sequential experience in the hippocampus during slow wave sleep. Neuron (2002) 36:1183–94. 10.1016/S0896-6273(02)01096-6 12495631

[75] Marshall L Born J. The contribution of sleep to hippocampus-dependent memory consolidation. Trends Cogn Sci (2007) 11(10):442–50. 10.1016/j.tics.2007.09.001 17905642

[76] Cartwright RD The Twenty-Four Hour Mind: The Role of Sleep and Dreaming in Our Emotional Lives. New York: Oxford University Press (2010).

[77] Barrett D Trauma and Dreams. Cambridge, MA: Harvard University Press (1996).

[78] Schreuder BJN Igreja V van Dijk D Kleijn W Intrusive re-experiencing of chronic strife or war. Adv Psychiatr Treat (2001) 7:102–8. 10.1192/apt.7.2.102

[79] Payne JD Stickgold R Swanberg K Kensinger EA. Sleep preferentially enhances memory for emotional components of scenes. Psychol Sci (2008) 19(8):781–8. 10.1111/j.1467-9280.2008.02157.x 18816285 PMC5846336

[80] Walker MP van der Helm E. Overnight therapy? The role of sleep in emotional brain processing. Psychol Bull (2009) 135(5):731–48. 10.1037/a0016570 19702380 PMC2890316

[81] Domhoff GW Meyer-Gomes K Schredl M Dreams as the expression of conceptions and concerns: a comparison of German and American College Students. Imagination Cogn Pers (2006) 25:269–82. 10.2190/FC3Q-2YMR-9A5F-N52M

[82] Foulkes D Schmidt M Temporal sequences and unit composition in dream reports from different stages of sleep. Sleep (1983) 6(3):265–80. 10.1093/sleep/6.3.265 6622882

[83] Horton CL Memory consolidation in sleep: implications from dream science. Int J Dream Res (2012) 5(Suppl 1):S46–7.

[84] Kirov R Kolev V Verleger R Yordanova J. Labile sleep promotes awareness of abstract knowledge in a serial reaction time task. Front Psychol (2015) 7(6):1354. 10.3389/fpsyg.2015.01354 26441730 PMC4561346

[85] Yordanova J Kirov R Kolev V Increased performance variability as a marker of implicit/explicit interactions in knowledge awareness. Front Psychol (2015) 23(6):1957 10.3389/fpsyg.2015.01957 PMC4688353 26779047

[86] Saunders D Clegg H Roe CA Smith GD Exploring the role of need for cognition, field independence and locus of control on the incidence of lucid dreams during a 12 week induction study. Dreaming (2017) 27(1):68–86, 1053–797. 10.1037/drm0000044

